# Nephroprotection of Wood Apple (*Limonia acidissima*), Water Spinach (*Ipomoea aquatica*), and Moringa (*Moringa oleifera*) on Gentamicin-Induced Nephrotoxicity and Oxidative Stress in Rat Model

**DOI:** 10.1155/jnme/3688503

**Published:** 2025-07-16

**Authors:** Mousumi Akter, Sneha Sarwar, Maisha Majid, Mahbub Zaman Mithun, Badhan Banik, Md Saidul Arefin, Sheikh Nazrul Islam

**Affiliations:** ^1^Institute of Nutrition and Food Science, University of Dhaka, Dhaka 1000, Bangladesh; ^2^Department of Nutrition and Food Engineering, Daffodil International University, Savar, Dhaka 1216, Bangladesh

**Keywords:** antioxidant foods, functional foods, moringa, nephroprotection, nephrotoxicity, oxidative stress, water spinach, wood apple

## Abstract

**Objective:** The present research investigated the pharmacological effectiveness of three functional foods—wood apple (WA), water spinach (WS), and moringa (MO)—against gentamicin (GM)-induced nephrotoxicity and oxidative stress in rat models.

**Methodology:** The study was conducted on rat model. Twenty-five healthy Long Evan rats of both sexes were equally divided into five groups, which were studied for 7 days. GM at a dose of 80 mg/kg body weight was given daily intraperitoneally to rats of all groups except the normal control (NC). Simply, the NC and negative control (GM) groups received only regular diet. The 3 treatment groups received 20 g/rat/day of mashed WA, fried WS, and roasted MO with regular feed diet at 1:1 ratio. On the last experimental day (8^th^ day), all the rats were sacrificed to collect blood and kidney samples. Nephrotoxicity was assessed by biochemical estimation of serum creatinine (CK) and blood urea nitrogen (BUN), and oxidative stress was analyzed by determination of serum malondialdehyde (MDA) and superoxide dismutase (SOD) levels. In addition, histopathology of kidney tissue was also performed for final observation.

**Results:** By lowering uremic toxin (serum CK and urea) levels, all the three functional foods significantly (*p* < 0.05) improved kidney function and the GM-induced oxidative stress. However, the difference in the blood SOD level was found to be statistically insignificant (*p* > 0.05), nevertheless. The histopathological results in those groups corroborated the biochemical results of the food intervention groups.

**Conclusion:** The present attempt shows that consuming the foods containing antioxidant phytochemicals may be a possible way to combat nephrotoxicity and oxidative stress. Nonetheless, the dosage response of these functional foods and mechanism of action to nephroprotection need to be investigated.

## 1. Introduction

One important function of kidney is to rid the body of waste materials that are either ingested or produced by metabolism. Any form of renal damage caused directly or indirectly by drugs (overdose, drug–drug interactions, or side effects), acute kidney failure, tubulopathies, obesity, diabetes, hypertension, and so on are all examples of nephrotoxicity [1]. Acute kidney injury (AKI) causes complications in 5% of hospital admissions and 30% of ICU admissions [2]. Beside this, chronic kidney disease (CKD) is an important contributor to morbidity and mortality associated with noncommunicable diseases [3]. According to the 2010 Global Burden of Disease study, CKD rose in the global ranking of causes of death from 27th place in 1990 to 18th place in 2010, over a period of 2 decades [4]. The role of drug-induced nephrotoxicity in kidney illness, such as AKI, is becoming more widely acknowledged. Certain nephrotoxic medicines are more likely to cause kidney harm when taken in high doses or over longer periods of time [5]. Due to relatively large blood flow (20% of stroke volume) and the ability to extract and concentrate hydrosoluble toxic molecules, the kidney is prone to drug-induced damage [6]. Therapeutic agents that commonly cause nephrotoxicity are aminoglycosides antibiotics, amphotericin B, cisplatin, esthetic agents, and NSAIDs [7]. General mechanisms of drugs that cause nephrotoxicity include changes in glomerular hemodynamics, tubular cell toxicity, inflammation, crystal nephropathy, rhabdomyolysis, and thrombotic microangiopathy [8]. Aminoglycoside type antibiotic gentamicin (GM) is one of the leading causes of drug-induced nephrotoxicity [6].

All inflammatory diseases (arthritis, vasculitis, glomerulonephritis, lupus erythematosus, and adult respiratory diseases syndrome), ischemic diseases (heart diseases, stroke, and intestinal ischema), hypertension and preeclampsia, neurological disorders, smoking-related diseases, and many others are now thought to be influenced by oxidative stress [9]. The kidneys are negatively affected by oxidative stress mainly because of the fact that reactive oxygen species (ROS) production induces the recruitment of inflammatory cells and proinflammatory cytokine production, leading to an initial inflammatory stage [10].

Phytochemicals or herbal medications can be generated from numerous natural sources. These include a variety of functional foods, which are now widely employed to treat a variety of ailments, and this treatment technique is gaining in favor around the world [11]. Plant phytochemicals (small molecules generated from plants) are non-nutritive plant compounds with anti-inflammatory and disease-preventive activities [12]. They have a role in oxidative stress metabolism, which is important for the development and prevention of a wide range of chronic diseases [13]. Antioxidants aid to prevent damage during oxidative stress by eliminating potential oxidants or converting them into less reactive molecules [14]. Several antioxidant phytochemicals have been discovered to have anti-inflammatory effects, such as suppression of prostaglandin production, enzyme inhibition, and nuclear factor-kB activity, as well as an increase in cytokine production [15]. Moreover, they can reduce oxidative stress by lowering ROS production, reducing lipid peroxidation, acting as an effective free radical scavenger, raising GSH and NADP levels, and increasing the catalase, glutathione reductase, and superoxide dismutase (SOD) activity^∗∗∗^. The nephroprotective role of phytochemical containing foods such as wood apple (WA), water spinach (WS), and moringa (MO) were reported in several papers. It is reported that all of the food extracts have a significant effect on cardiac health [16, 17].

However, to the best of our research findings, the protective effect of WA, WS, and MO in their regular consumable form was not yet investigated and because of their high antioxidant qualities, this is the first trial to use WA, WS, and MO in their edible forms (mashed WA, fried WS, and dried MO leaves) to treat nephrotoxicity.

Nephrotoxicity has been stimulated by administrating a selective accumulation of GM in the renal cortex [18]. GM, an aminoglycoside, is the most commonly used antibiotic and very effective in treating life-threatening Gram-negative infections such as meningitis, endocarditis, infected burns, wounds, or skin lesions [19]. Preventing measures of GM-induced nephrotoxicity include single daily dosing (Bennett et al, 2009), administration during period of activity, intake of antioxidants, vit-C, and vit-E, and hemodynamic control [20]. The use of several compounds with antioxidant activity has been successfully used to prevent or ameliorate GM-induced nephrotoxicity [21]. Hence, the current research has come to an end to evaluate the nephroprotective effect of the selected phytochemical-containing foods (WA, WS, and MO) on GM-induced nephrotoxicity in animal models.

## 2. Materials and Methods

### 2.1. Animals, Acclimatization, and Ethics

The study was carried out in the Institute of Nutrition and Food Science (INFS), University of Dhaka. A total of 25 healthy long Evan rats weighing 242 ± 20 g were collected from the animal house of the Pharmacy Department, Jahangirnagar University, Savar. All the rats were kept in ambient condition in well light and well-ventilated animal house of the INFS, University of Dhaka. The environment of the animal house was properly maintained because it was essential for animals' wellbeing, the quality of animal research, and also the health and safety of the investigator. Each rat was housed in separated similar metallic cages. The room was well ventilated and maintained under constant 12 h light and 12 h dark cycle, a temperature of about 26–28°C, and 45 ± 5% humidity. The cages were cleaned regularly and proper hygienic and sanitary measures were ensured and inspected daily during the experiment. Waste product was removed regularly. The rats were acclimatized for 14 days. During this period, they were given ad libitum with the standard laboratory diet except during the day of sacrifice and collecting blood samples when they were kept overnight fasting. Animals were handled following the protocol sets provided by the Swiss Academy of Medical Sciences (SAMS) and the Swiss Academy of Sciences (SCNAT). Moreover, all the experimental protocols and procedures were approved by the Ethical Review Committee Faculty of Biological Science, University of Dhaka (Ref. no. 118/Biol.Scs.).

### 2.2. Collection and Preparation of Food Samples

Food samples were selected as per following criteria: seasonal availability during experimental period, local acceptance and convenience, and their values of certain nutrients (high polyphenol content, rich source of antioxidants, vitamin C, and high beta-carotene content). This primary selection was made by searching database of the food composition table for Bangladesh [22] and the food composition table for India [23]. From these databases, two leafy vegetable sample: WS(*Ipoema aquatic*) and MO leaves (*Moringa Oleifera*) and one fruit sample: WA (*Limonia acidisima*) were chosen for the investigation. All three of the samples were purchased from Sutrapur Bazar, Old Dhaka, Bangladesh.

Before cooking, the leaves of WS were thoroughly washed, chopped, and then fried. In a frying pan, 100 g WS was fried in 10 mL hot refined soybean oil until the sample became crisp-tender [24]. The fried sample was mixed with the regular rat diet at 1:1 ratio and then administered to the rats. For WA, the edible portion was removed from it and mashed in mortar and pestle. The mashed sample was mixed with the regular diet at 1:1 ratio and administered to the rats. In case of moringa, the leaves were cleaned and got ready to dry. The leaves were cabinet dried at 50° C for 4 h to make powder of these [25]. Then, the dried leaves were made to powder through the mortar and pestle. This powder was then mixed with the regular diet at 1:1 ratio and administered to the rats.

A preliminary pilot test was done before on rats to examine the consumption habits of the tested items. Besides, the doses for these interventions were determined based on the previous survey, which also evaluated the potential of similar antioxidant-rich foods in animal models [1]. According to these evidences, the rats were given the selected foods in amount of 20 g/day with regular feeding in their regular consumable form, maintaining translational relevance for human consumption.

### 2.3. Drugs and Chemicals

Commercial GM injection (total 45 ampoules (3500 mg), 80 mg per ampoule) was collected from a model pharmacy, Square Pharmaceuticals Ltd (Dhaka, Bangladesh). Laboratory standard thiobarbituric acid, trichloroacetic acid, hydrochloric acid, 10% formalin, 0.9% saline, phosphate buffer, detergent, uricase, dichlorophenol sulphonate, ascorbate oxidase, peroxidase, aminoantipyrine, urease enzyme reagent, EDTA, sodium salicylate, sodium nitroprusside, alkaline hypochlorite, sodium hypochlorite, sodium hydroxide, urea standard, picric acid, creatinine standard, Nitrite standard solution, Griess reagent, and alcohol were purchased from Sigma-Aldrich (St. Louis, MO, USA) and used during the research.

### 2.4. Experimental Design

After acclimatization for 14 days, the rats were divided randomly into five groups, containing five rats in each group. The study lasted for 7 days. Throughout the study period, all animals except the normal control (NC) group were subjected to nephrotoxicity by administering GM 80 mg/kg/day intraperitoneally [1]. The following were the groups:

 
*Group I* (NC): This group of animals were given the standard laboratory diet only throughout the course of experiment.
 
*Group II* (experimental control [GM group]): Animals were made nephrotoxic by GM at the dose of 80 mg/kg body wt/day and fed the standard diet only.
 
*Group III* (intervention group-A: GM + WA): In this group, the laboratory diet was mixed with mashed WA in a 1:1 ratio (laboratory diet: WA). :Each rat received about 20 g mashed WA per day for 7 days.
 
*Group IV* (intervention group-B: GM + WS): In this group, the laboratory diet was mixed with fried WS in a 1:1 ratio (laboratory diet: WS). This group was also given 20 g WS daily 7 days.
 
*Group V* (intervention group-C: GM + M): In this group, the laboratory diet was mixed with dried MO leaves in a 1:1 ratio (laboratory diet: M). This group was also given 20 g MO daily for 7 days.

### 2.5. Collection of Blood Sample and Serum Separation

Blood collection procedures were conducted following ethical guidelines for animal research and complied to the principles outlined by Institutional Animal Ethics Committee (IAEC) and the National Research Council's Guide for the Care and Use of Laboratory Animals [26]. Animals were sacrificed under chloroform (30%) anesthesia after 7 days of therapy (day 8^th^), and using sterile disposable syringes, approximately 3–4 mL of blood samples from each rat were collected in clean and dry test tubes through cardiac puncture with proper identification numbers. The serum was separated by centrifugation at 3000 rpm for 15 min and collected by micropipette and transferred to the labeled Eppendorf tubes after the blood samples had clotted for 45 min. All these serum tubes were preserved in a refrigerator at −18°C for biochemical analysis (determination of serum creatinine, blood urea nitrogen (BUN), malondialdehyde (MDA), and SOD activities).

### 2.6. Collection of Kidney Sample for Histopathological Examination

The experimental protocol for tissue collection was approved by Ethical Commiittee of Faculty of Life Sciences, University of Dhaka. All procedures were conducted following ethical guidelines of animal research to minimize pain and distress. From each rat, both left and right kidneys were collected and washed with 0.9% saline water, wiped with tissue paper, weighed, and recorded. The weights were measured by an electric balance analyzer (Mettler Toledo, Zurich, Switzerland). The kidney samples were preserved in 10% neutral buffered formalin and stored at −18°C. Then, they were taken to animal cell of BCSIR on the next day and then undergo dehydration, clearing, paraffin infiltration, embedding, section cutting, and staining for histological examinations. 2.6 Estimation of nephrotoxicity marker.

The kidney function was estimated by measuring serum creatinine (CK) and BUN. These parameters of rats were analyzed with a semiauto biochemistry analyzer (AUSTRIA). Serum CK was determined by the kinetic method without deproteinization–Jaffle reaction and expressed as mg/dL [27]. Serum urea was quantitatively estimated by urease/salicylate colorimetric method endpoint [28]. Both serum CK and serum urea were expressed as mg/dL during statistical analysis.

### 2.7. Estimation of Stress Biomarkers

Serum MDA level and SOD were analyzed by a semiauto analyzer. Estimation of MDA was done by the thiobarbituric acid assay method [29]. Serum Cu–Zn containing SOD activity was enumerated spectrophotometrically [30].

#### 2.7.1. MDA Measurement

The level of MDA was measured by the thiobarbituric acid assay method [29]. In this method, 15% w/v trichloroacetic acid, 0.375% w/v thiobarbituric acid, and 0.25M hydrochloric acid were used as reagent (TCA–TBA–HCl). The TBA was obtained from Sigma-Aldrich (Product no.: 5500-25G, P.Code: 376796583, Lot no: SKB5697354) and TCA from Merck (Product name: Trichloroacetic acid, Likely Batch no: 82234205001730). The 1.0 mL of biological sample was combined with 2.0 mL of TCA–TBA–HCl and mixed thoroughly. The solution was heated for 15 min in a boiling water bath. After cooling, the flocculent precipitate is removed by centrifugation at 1000 g for 10 min. The absorbance of the sample was determined at 535 nm against a blank that contains all the reagents accept the biological sample.

#### 2.7.2. SOD Measurement

SOD activity was determined spectrophotometrically, based on the ability of the enzyme to inhibit the autooxidation of pyrogallol [30]. The autooxidation of pyrogallol in the presence of EDTA in the pH 8.2 was 50%. The principle of this method was based on the competition between the pyrogallol autooxidation by O_2_^−^ and the dismutation of this radical by SOD [31]. The absorption was read at the wavelength of 420 nm against Tris-EDTA buffer at zero time and after 1 min of the addition of pyrogallol.

### 2.8. Preparation of Kidney Specimen for Histopathological Examination

Sections were cut using a microtome after the kidneys had been collected and preserved properly. They were then treated and embedded in paraffin wax. After that, sections were viewed under a light microscope after being stained with eosin and hematoxylin [32]. Histopathological lesions were evaluated and assigned scores as follows [33]:

  Score 0 = normal.
  Score 1 = no glomerular and interstitial inflammation of inflammatory cells and few congested blood vessels in the interstitium (< 1% of total tubular population).
  Score 2 = tubular epithelial necrosis and desquamation easily seen but involving less than half of cortical tubules.
  Score 3 = more than half of proximal tubules showing desquamation of necrosis, moderate glomerular and interstitial infiltration of inflammatory cells and.
  Score 4 = complete or almost complete tubular necrosis.

### 2.9. Photography

Photographs were taken from the representative sections of each group using a camera fitted with microscope.

### 2.10. Statistical Analysis

The obtained data were statistically analyzed, and the results were expressed as means ± standard deviation (SD). Differences between groups were assessed using a one-way analysis of variance (ANOVA). The Tukey test was conducted for multiple comparisons when group difference was significant. Paired *t*-test was conducted to observe the significant bodyweight variation due to the administration of drugs. Results were considered statistically significant at *p* < 0.05. All the analyses were run using the SPSS 25.0 for Windows.

## 3. Results

### 3.1. Effect of GM on Body Weight and Kidney Weight

The body weight variation in the five different groups of rats was observed by comparing the weights measured at the beginning and the end of the study.  Table 1 illustrates that the rats in the GM group significantly reduced their body weight (initial weight vs. final weight: 246 g vs. 231 g; *p*=0.012). However, the rats within the NC group and WS exhibited a notably elevated bodyweight (initial weight vs. final weight, NC: 248 g vs. 278 g; *p*=0.001 and WS: 246 g vs. 273 g; *p*=0.003).

As illustrated in Table 2, treatment with GM groups of rats showed an increase in kidney weight when compared with other groups of rats but no significant change was observed among the groups.

### 3.2. Effect of GM on Biochemical Parameters

Upon infusion of GM alteration in two biochemical parameters (serum CK and BUN) were observed in different groups of rats.  Table 3 shows the effect of GM and the combination of different food items. Serum CK level (mean ± SD) was significantly lower in all intervened groups (GM + WA: 1.70 ± 0.204, GM + WS: 0.94 ± 0.080, GM + MO: 0.98 ± 0.114; *p* < 0.05) of rats compared with the corresponding GM (1.70 ± 0.204) group of rats (*p* < 0.05). In terms of the BUN level, all three intervened groups also showed significantly low concentration (GM + WA: 74.60 ± 18.02, GM + WS: 59.2 ± 21.32, GM + MO: 64.0 ± 17.56) than the only GM group (83.80 ± 31.73 mg/dL) of rats (*p* < 0.05).

Along with these renal toxicity biomarkers, a significant alteration was observed in stress parameter (MDA) also. The MDA level was significantly (*p* < 0.05) lower in all the treatment groups (8.15 ± 1.46 to 9.48 ± 1.17 μmol/L), including the NC group lower (7.42 ± 0.89 μmol/L) than the GM group (16.05 ± 4.04 μmol/L) (Table 3). However, in case of SOD level, no significant difference was observed among the treatment groups and GM control group.

### 3.3. Histopathological Analysis

**Figure 1:**
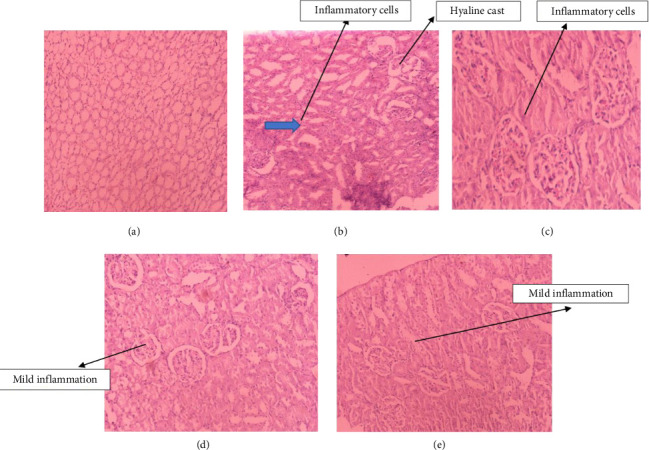
Histopathology of kidney cell: (a) normal control (NC) group, (b) negative control (GM) group, (c) wood Apple (GM + WA) group, (d) water spinach (GM + WS) group, and (e) moringa (GM + MO) group (arrow indicates hyaline cast; H & E staining and 20× magnification were applied to the sections).

The observations from the histopathology analysis of the kidney tissues were in agreement with the results of the biochemical parameters. In Figure 1(a), the NC group's kidney tissues showed normal glomerular structure and interstitium and no inflammatory cell infiltration (Score 0). The kidney tissues of the GM group exhibited extensive necrosis, glomerular congestion, interstitial congestion, and inflammatory cell infiltration (Figure 1(b); Score 4). The WA (GM + WA) group showed no significant improvement in kidney tissues. Extensive necrosis, glomerular congestion, interstitial congestion, and inflammatory cell infiltration were found in the WA group (Figure 1(c); Score 4). The kidney tissues, glomerular, and interstitial infiltration of inflammatory cells of the GM + WS group showed significant improvement in the pathological features that were focally present along with mildly congested blood vessels in the interstitium, mild hyelin cast in the tubule (Figure 1(d); Score 2). The kidney tissues of the MO (GM + MO) group revealed mild desquamation of proximal tubules. Focal glomerular and interstitial infiltrations of inflammatory cells along with mildly congested blood vessels in the interstitial mild hyelin cast in the tubule were found (Figure 1(e); Score 3).

## 4. Discussion

The aim of the present study is to explore the effects of three antioxidants-rich foods (WA, WS, and MO) to ameliorate GM induced nephrotoxicity and oxidative stress. The cotreatment of these foods with GM for 7 days significantly improved renal function by reducing serum CK and BUN levels and oxidative stress marker (MDA level). We have used Long Evan rats for this experiment prior to human trials; animal research offers proof of an intervention's effectiveness and reduces its unintended danger. Rats are typically employed in nephrotoxicity investigations [34].

In this study, we observed significant physiological changes and various alterations in the anatomy during the bodyweight variation test. Body weight declined considerably in all rat groups over the study period as compared with the NC group. When GM-injected rats were cotreated with WA, WS, and MO, their body weight tended to increase relative to the GM negative control group, but this impact was only statistically significant for WS intervened group. The weight reduction in GM-treated negative control group is higher. This might have happened due to the loss of skeletal muscle and lean body mass [35]. The significant and progressive weight losses in GM-treated rats are also reported in some studies [36]. Beside this, it has been found that the GM group has increased kidney weight compared with the NC group. Research indicates that infusion of chemical (GM) might have increased the kidney weight due to its damaging effects on renal tissues [37]. But the weight is decreased in all the treatment groups probably due to the removal of chemical from kidney from the effect of the food that served to restore kidney functionality [38].

Significant renal effects were observed in this investigation following GM administration, as evidenced by a notable rise in stress and renal function indices as well as histological alterations. In GM-induced nephrotoxicity, substantial increases in blood CK and urea concentration were proposed as a major functional impairment of the kidney [39]. This study builds on previous research demonstrating the negative effects of GM on CK, BUN, MDA, and SOD levels [37]. The significant rise in CK and BUN is in line with earlier studies that demonstrate nephrotoxicity and skeletal muscle damage caused by GM [40, 41]. Furthermore, the increase in MDA, a marker of lipid peroxidation, and the decrease in SOD, an antioxidant enzyme, support the oxidative stress associated with GM therapy [42, 43]. The increased production of free radicals in GM-induced nephrotoxicity is shown by the higher amount of MDA. Rats that were concurrently treated with WA, WS, MO, and GM showed a substantial inhibition of the increase of serum CK, BUN, and MDA as compared with animals who were just treated with GM. Other researchers who used various antioxidants also reported similar findings [44, 45].

The study highlights the antioxidant and nephroprotective properties of *Limonia acidissima* (WA), *Ipomoea aquatica* (WS), and *Moringa oleifera* (MO). WA extract, rich in various compounds including thymol, exhibited strong free radicals scavenging activity, reducing ROS generation [46, 47]. It also showed nephroprotective effects, reducing BUN and serum CK levels [48]. WS, a good natural antioxidant source, regulates detoxification enzymes and exhibits significant scavenging effects on nitric oxide formation [49, 50]. Boiling or frying WS enhances its radical scavenging ability, positively impacting renal and stress function parameters [1, 24]. Four likely arguments were put out by Morales and Babbel as to why cooking increases the antioxidant activity of vegetables: the thermal breakdown of cell walls lead to (1) the release of antioxidant components; (2) production of stronger antioxidants; (3) inactivation of oxidative enzymes; and (4) synthesis of new antioxidants [51]. Previous research at the same institution of University of Dhaka has also shown improved renal function with boiled WS, supporting the current findings [1]. Similarly, *Moringa oliefera* leaves, abundant in polyphenols and phenolic acids, demonstrated significant nephroprotective effects by reducing oxidative stress markers and improving renal function [52, 53]. Our study aligns with the previous research, showing significantly lower levels of serum CK and BUN in the moringa group. The previous studies by Stephen Akinrinde et al. [16] and Ouédraogo et al. [54] also revealed the significant same decreased BUN and serum CK level as ours. These may lead one to conclude that these plants as dietary supplement can mitigate renal damage and oxidative stress.

A previous study by Sarwar et al. investigated the nephroprotective effects of red grape and WS alongside probiotics in GM-induced nephrotoxicity in rats [1]. While the present study shares a similar experimental model (GM-induced nephrotoxicity in long Evan rats), the intervention foods and their processing forms are notably different. In this study, we explored the protective potential of fried WS, mashed WA, and roasted MO leaves powder, each selected for their distinct phytochemical profiles and traditional dietary usage. Moreover, unlike the previous study, our intervention included processed forms of antioxidant-rich foods, aiming to mimic more realistic human consumption patterns. The differentiation enhances the translational value of our findings and provides novel insights into the renoprotective efficacy of regionally consumed antioxidant-rich foods.

However, by examining the preventive effects of natural substances—WA, WS, and MO—against GM-induced biochemical changes, this work fills a critical research vacuum. All of the tested foods were ingested to rats in their regular consumable form rather than following any extraction procedure. It is due to establish their easiest way of normal daily consumption pattern for human being. This new understanding advances our understanding of therapies aimed at mitigating the effects of GM. Therefore, according to the findings of this study, WA, WS, and MO in their regular consumable form might be an excellent way to reverse oxidative stress, hence providing nephroprotection.

### 4.1. Strength and Limitation of the Study

The primary advantage of the research lies in its utilization of whole, readily consumable food items rather than extracts. The chosen foods are abundant in phytochemicals, demonstrating their potential to mitigate nephrotoxicity and are readily accessible in the local context.

While the study yielded a significant result, certain constraints were also identified. For instance, a substantial sample size is typically essential for achieving more robust results, but our limited funding prevented us from obtaining a larger sample. In addition, the study's duration was brief, lasting only 7 days excluding the acclimatization period, which proved inadequate for capturing all anatomical changes associated with nephrotoxicity. Furthermore, the optimal dosage for the selected food items was not established. In addition, the estimation of another parameter related to kidney toxicity, such as uric acid, oxidative stress parameters in kidney tissue, and image-based quantification for histopathological lesion scoring were unfeasible due to its high cost.

### 4.2. Future Research

WA, WS, and MO exhibit potential as nephroprotective agents for mitigating nephrotoxicity by effectively restoring nephrotoxic biomarkers and reducing oxidative stress levels back to normal. Further inquiry is imperative to validate the notion of augmenting the consumption of these specific foods to enhance nephrotic wellbeing. It is essential to delve into the mechanisms through which antioxidant intake impacts renal health. In addition, additional research is warranted to ascertain the necessary dosage and optimal strategies for enhancing renal health.

## 5. Conclusion

The animal model of nephrotoxicity induced by GM intraperitonially was successful, which mimic similar studies previously done.

WA, WS, and MO exhibited excellent nephrotoxicity markers (BUN and serum CK) attenuating properties. These foods also significantly reduce oxidative biomarker such as the MDA level by their free radicals scavenging property. No significant result was found in the case of SOD activity. Thus, these foods are proven to have good nephroprotective property along with oxidative stress-reducing activity due to their antioxidant, anti-inflammatory, and antiapoptotic properties. In terms of alleviating serum biomarkers, WS showed the best effect by reinstating the serum biochemical parameter near to normalcy: WA and MO also showed significant result.

All chosen food samples have shown their excellent capacity in preventing GM-induced nephrotoxicity in Long Evan rats. This study suggests that the tested foods can be good choices to combat nephrotoxicity occurred in GM ingestion. However, further studies to elucidate more of their mechanism and potentials are advised.

## Figures and Tables

**Table 1:** Effects of gentamicin, wood apple, water spinach, and moringa on body weight of rats.

Groups	Initial weight (g) Mean ± SD	Final weight (g) Mean ± SD	Mean difference (final-initial)	Paired *t*-test	*p* value
Normal control (NC)	248.4 ± 20.51	278.00 ± 21.07	29.6	−9.68	**0.001**
Gentamicin (GM)	246.0 ± 24.30	231.60 ± 25.7	−14.4	4.34	**0.012**
Wood apple (GM + WA)	241.6 ± 41.6	250.40 ± 43.0	8.8	−0.83	0.456
Water spinach (GM + WS)	246.4 ± 38.17	273.20 ± 40.46	26.8	−6.65	**0.003**
Moringa (GM + MO)	242.8 ± 46.92	246.00 ± 40.25	3.2	−0.691	0.528

*Note:* The bold format indicated the significant level (*p* < 0.05).

**Table 2:** Effect of gentamicin, wood apple, water spinach, and moringa on relative kidney weight.

Groups	Body weight (g) Mean ± SD	Kidney weight (g) Mean ± SD	Kidney/body ratio (g/g) Mean ± SD
Normal control (NC)	278.00 ± 21.07	0.682 ± 0.28	0.0024 ± 0.0008
Gentamicin (GM)	231.60 ± 25.7	1.128 ± 0.23	0.0048 ± 0.0009
Wood apple (GM + WA)	250.40 ± 43.0	1.019 ± 0.07	0.0041 ± 0.0006
Water spinach (GM + WS)	273.20 ± 40.46	0.946 ± 0.29	0.0034 ± 0.0008
Moringa (GM + MO)	246.00 ± 40.25	1.110 ± 0.15	0.0046 ± 0.0007

*Note:* Kidney versus body weight ratio, g/g.

**Table 3:** Effect of gentamicin, water spinach, wood apple, and moringa on serum biochemical parameters.

Groups	Biochemical parameters			
	CK (mg/dL)	BUN (mg/dL)	MDA (μmol/L)	SOD (U/mL)
Normal control (NC)	0.54 ± 0.096^∗^	19.0 ± 1.87^∗^	7.42 ± 0.89^∗^	2.96 ± 0.60
Gentamicin (GM)	1.70 ± 0.204^∗^	83.80 ± 31.73^∗^	16.05 ± 4.04^∗^	2.60 ± 0.16
Wood apple (GM + WA)	1.28 ± 0.205^∗^	74.60 ± 18.02^∗^	9.48 ± 1.17^∗^	2.75 ± 0.50
Water spinach (GM + WS)	0.94 ± 0.080^∗^	59.2 ± 21.32^∗^	8.15 ± 1.46^∗^	3.55 ± 0.84
Moringa (GM + MO)	0.98 ± 0.114^∗^	64.0 ± 17.56^∗^	9.00 ± 1.66^∗^	2.99 ± 0.34

*Note:* Values are expressed as the mean ± SD.

^∗^Significance level *p* < 0.05.

## Data Availability

All the obtained data of this research are included within the article and if any data supporting the findings of this study needed, they are available from the corresponding author upon reasonable request.
